# Genetic pre-screening for glaucoma in population-based epidemiology: protocol for a double-blind prospective screening study within Lifelines (EyeLife)

**DOI:** 10.1186/s12886-020-01771-9

**Published:** 2021-01-07

**Authors:** Anna Neustaeter, Ilja Nolte, Harold Snieder, Nomdo M. Jansonius

**Affiliations:** 1 Department of Ophthalmology, University of Groningen, University Medical Center Groningen, P.O.Box 30.001, 9700 RB Groningen, Netherlands; 2 Department of Epidemiology, University of Groningen, University Medical Center Groningen, Groningen, The Netherlands; 3 Graduate School of Medical Sciences (Research School of Behavioural and Cognitive Neurosciences), University of Groningen, Groningen, The Netherlands

**Keywords:** Genetic risk score, Glaucoma, Screening, Prospective design, Lifelines

## Abstract

**Background:**

Early detection of glaucoma is paramount to maintain patients’ eyesight, however glaucomatous vision loss tends to begin in the periphery with up to 50% of patients unaware they are affected. Because glaucomatous vision loss is permanent, screening appears attractive, but currently is not cost-effective. Therefore we aim to investigate the utility of genetic pre-screening for glaucoma in a population-based setting, called EyeLife.

**Methods:**

EyeLife adopts a double blind prospective design with contrasting groups. Selected participants (*n* = 1600) from the Lifelines cohort are 55 years of age or older, and of either the highest or lowest 20% of the genetic risk distribution for glaucoma. We obtained a highly curated list of genetic variants from the literature to obtain each participants’ genetic risk for glaucoma. Participants will undergo comprehensive ophthalmic screening. The primary outcome is the relative risk of glaucoma given a high genetic risk compared to a low genetic risk.

**Discussion:**

If genetic pre-screening is successful, it will increase the yield of a glaucoma screening program by focusing on high-risk individuals. This, in turn, may improve long-term visual health of middle-aged and elderly people.

**Trial registration:**

Ethics approval was obtained on January 31, 2019, and the study was retrospectively registered with the Netherlands Trial Register (NL8718) on the 17th of June, 2020.

## Background

Age-related ocular disorders like glaucoma contribute greatly to the burden of healthcare and loss of quality of life in older populations [1, 2]. Glaucoma, a group of neurodegenerative diseases of the optic nerve, is the second-leading cause of irreversible vision loss in the world. Up to one half of those with glaucoma are unaware of their disease status [3]. Adult-onset primary open-angle glaucoma is the most common subtype in Caucasians and within this article “glaucoma” refers to this subtype. Initial glaucomatous visual field loss (GVFL) may not be perceived as the brain perceptually masks visual field defects with visual features of nearby regions [4, 5]. The slow course of the disease in combination with visual field defects initially in non-overlapping locations of each eye contribute to patient unawareness as well. Lowering the intraocular pressure [6–8](IOP), currently the only proven treatment modality, reduces end-of-life blindness from approximately 50 to 15% [6–8]. The combination of unawareness and the benefits of timely treatment suggest a pivotal role for screening.

Historical glaucoma screening programs were based exclusively on IOP measurements. However, only ~ 10% of untreated ocular hypertensive patients develop glaucoma within 5 years [9]. Additionally, in populations screened for glaucoma, only a minority of newly diagnosed patients had IOP outside the normal range at the time of screening [10–12]. Diurnal IOP fluctuation may partially explain this, but glaucoma still develops in those with IOP consistently within normal limits, a condition termed ‘normal tension glaucoma’. Because IOP lowering is also effective in normal tension glaucoma, an increased vulnerability of the optic nerve head (ONH) was hypothesized, with several reported factors contributing to increased vulnerability [13]. Other screening methods for glaucoma include testing functional vision via visual field (VF) testing, or assessing structural damage to the optic nerve or retinal nerve fiber layer (RNFL), typically with optical coherence tomography (OCT). However, despite technological advancements and detailed analyses, the poor screening performance of these individual techniques still limits applicability in the general population, due to the low prevalence of glaucoma (~ 2% in those aged 55 and older) [14]. With such a low prevalence, a single screening test requires a specificity of at least 95–97.5% [15–17]. Using known glaucoma risk factors as a pre selection tool for screening aims to increase the prior probability of glaucoma in the population while limiting case-loss. People at an older age, of African descent, those with a family history of glaucoma, as well as those with myopia are at a higher risk of glaucoma [14, 18–20]. However, limiting glaucoma screening to only individuals with these risk factors is ineffective, as the majority of cases with one or more known glaucoma risk factors are detected in regular care [10, 21].

Accordingly, it is well-established that glaucoma and related endophenotypes are substantially heritable [22]. Inherited forms of glaucoma may be attributed to single gene mutations, like myocilin (*MYOC)* and optineurin (*OPTN*), however these cases are rare and tend to occur early in life [23]. For the majority of glaucoma cases, genetic susceptibility arises from the combined effects of a number of genetic variants, each with individually small effects. These genetic variants can be combined into a single genetic risk score (GRS). Genome-wide association studies (GWAS) continually uncover novel genetic variants, termed single nucleotide polymorphisms (SNPs), associated with not only glaucoma risk and progression, but also continuous glaucoma endophenotypes like IOP, optic nerve properties [disc area (DA), cup area (CA), cup-to-disc ratio (CDR)], central corneal thickness (CCT), retinal nerve fiber layer thickness (RNFLT), and peripapillary atrophy (PPA) [24–27]. A recent retrospective cross-sectional cohort study reported that individuals at the top decile of genetic risk are at a 15-fold increased risk in developing advanced glaucoma, compared to the bottom decile [26]. To date, the predictive ability of a glaucoma GRS used as a screening tool has not been validated [28] and the research question is: Are those at a higher genetic risk for glaucoma more likely to actually have glaucoma?

For this purpose, we aim to investigate the predictive ability of a glaucoma GRS. We extracted a highly curated list of SNPs that were genome-wide significantly associated with glaucoma and/or related-endophenotypes and combined them to create a glaucoma GRS. The GRS is employed in a population-based cohort as a pre-selection tool for glaucoma. We are currently conducting a double-blind prospective study with contrasting genetic risk groups to evaluate the feasibility of genetic pre-screening for glaucoma case detection. The primary outcome is the relative risk of glaucoma for individuals at the highest versus the lowest 20% of the genetic risk distribution.

## Methods and analysis

### Study design

This is a double-blind prospective study with contrasting genetic risk groups, investigating the predictive ability of a glaucoma GRS. It is based out of the ophthalmology department of the University Medical Center Groningen (UMCG), and called EyeLife. Participants are selected from Lifelines, a large prospective population-based cohort study of the Northern Netherlands [29]. It examines the health and health-related behaviours of 167,729 persons, in a unique three-generation design. Lifelines employs a broad range of investigative procedures in assessing the biomedical, socio-demographic, behavioural, physical, and psychological factors which contribute to the health and disease of the general population, with a special focus on multi-morbidity and complex genetics [30]. The cohort structure is described fully elsewhere [31]. Genetic information is currently available for more than 50,000 participants, and a subset of this population is invited to take part in EyeLife.

### Characteristics of participants

Lifelines participants at either the highest or lowest 20% of the glaucoma genetic risk distribution with signed informed consents are eligible to participate in EyeLife. In total, 1600 genotyped Lifelines participants 55 years of age and older, and amenable to additional research studies, are selected for participation in EyeLife.

### Genotype information

Genotypic information for Lifelines was obtained in two phases. Initially 15,638 unrelated individuals within Lifelines were genotyped using the Illumina CytoSNP 12 v2 array (Illumina, San Diego, CA, USA). Initial quality control was carried out in PLINK [32]. Markers with a call rate < 95%, Hardy-Weinberg equilibrium *p* < 0.0001, or minor allele frequency < 1% were excluded. Samples with sex-mismatches, deviating heterzygosity (> 4 standard deviations from the mean), non-European ancestry, a call rate of < 95%, duplicated samples, or first degree relatives were excluded. A total of 268,407 autosomal genetic markers, and 13,436 samples remained. Imputation was subsequently carried out using Beagle v3.1.0 [33] with the HapMap Phase 2 CEU reference panel (release 24, build 36) [34, 35].

Next, the UMCG Genetics Lifelines Initiative (UGLI) genotyped 38,030 additional Lifelines participants using the Infinium Global Screening Array® (GSA) MultiEthnic Disease Version. Quality control focused on the autosomal and X chromosomes (691,072 markers). First, duplicate markers were removed, then samples and markers of low quality were filtered using call rate thresholding in two stages (80 and 99%, respectively). Following that, variants that deviated significantly from Hardy-Weinberg equilibrium (*p* < 1 × 10^− 6^) or that were monomorphic (minor allele frequency = 0) were removed. Next, samples with a deviating heterozygosity (> 4 standard deviations from the expected mean adjusted for observed runs of homozygosity), unexpected duplicate samples, and samples of non-European ethnicity were excluded. After passing initial quality control, 36,339 samples and 571,420 genetic markers were imputed through the Sanger Imputation Service (http://imputation.sanger.ac.uk) utilizing the Haplotype Reference Consortium panel (http://www.haplotype-reference-consortium.org).

### Glaucoma genetic risk score

Genome-wide association analyses indicate which SNPs are associated with an outcome of interest; outcomes can be binary such as glaucoma, or continuous like IOP. As there are millions of regressions per GWAS, a global threshold of *p* ≤ 5*10^− 8^ was established for genome-wide significance [36]. A GRS is the genetic profile of SNPs that increase the risk of the disease or trait. It can be unweighted via counting the number of risk alleles, or weighted by the GWAS effect sizes of the SNPs included. The SNP effect direction is aligned so included SNPs represent an increased risk. The generalized GRS equation is:

1 $$ GRS={\varSigma}_{i=1}^N{\omega}_i\bullet {\chi}_i $$

where *N* is the number of SNPs in the GRS, *ω*_*i*_ is the weight for SNP *i* (in an unweighted GRS *ω*_*i*_ is 1; in a weighted GRS *ω*_*i*_ is the regression coefficient of SNP *i* derived from GWAS summary statistics with glaucoma as the outcome - we used the effect size with glaucoma as the outcome, even where the SNP was originally genome-wide significant for an endophenotype), and *χ*_*i*_ is the allelic dosage of the risk variant *i* [35]. We created and employed a weighted GRS as they explain more variance in complex disorders like glaucoma [35].

The glaucoma GRS used in this study is novel as it utilizes genome-wide significant SNPs from both glaucoma in addition to related endophenotypes to create a highly curated glaucoma GRS. We aim to improve upon current SNP-based glaucoma risk prediction by incorporating endophenotype-associated SNPs and applying the GRS prospectively in a population-based setting as a pre-screening tool. What follows below are the details of the approach.

**Fig. 1:**
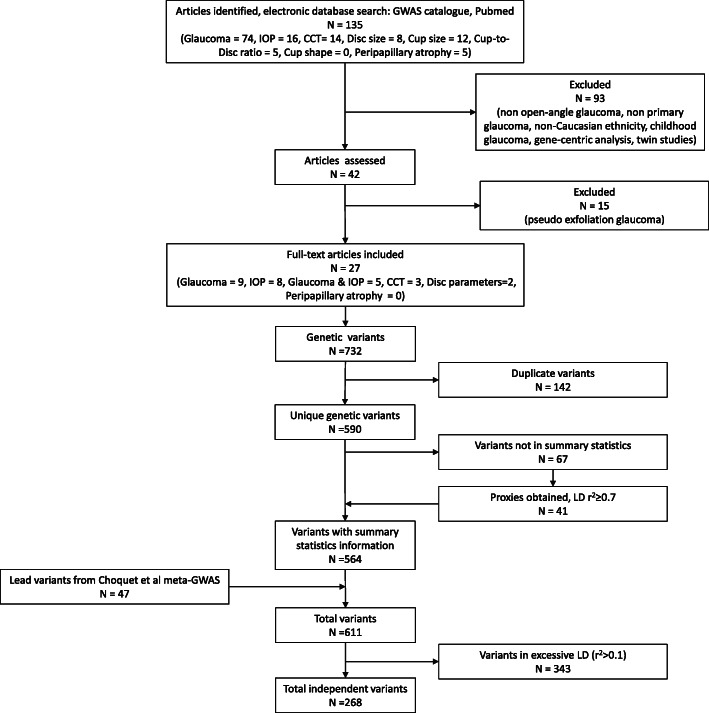
Glaucoma genetic risk score flow diagram. The decision tree shows the article and genetic variant selection to obtain relevant SNPs for the glaucoma GRS

A literature search was performed on November 8th, 2018 for GWAS of glaucoma and relevant endophenotypes (IOP, CCT, optic disc parameters, RNFL, and PPA) in populations of European ancestry, see Fig. 1 for a flow diagram of article and SNP selection. Initially, 134 articles were obtained. Articles were excluded (*n* = 93) for the following reasons: duplicates, the outcome was not primary/open-angle glaucoma, the population was not of European ancestry (mixed ethnicity GWAS were permitted if European ancestry was included), the GWAS was performed in children, the study design was gene-centric study designs, or it was a twin study. The full text of 42 articles was assessed, and articles regarding pseudoexfoliation glaucoma (*n* = 15) were further excluded. From these 27 articles, 732 variants were genome-wide significantly associated (*p* ≤ 5 × 10^− 8^) with glaucoma or related endophenotypes. Duplicated SNPs (*n* = 142) were excluded, resulting in 590 SNPs. Next, the 590 SNPs were looked up in the summary statistics of Choquet et al. [27], a large glaucoma meta-GWAS combining the UK Biobank and GERA cohorts, to obtain SNP effect sizes. We always utilized the effect size from the glaucoma meta-GWAS, even where the SNP was originally genome-wide significant for an endophenotype. Of the 590 SNPs, 67 lacked summary statistic information, we obtained proxies using a linkage disequilibrium (LD) threshold of r^2^ ≥ 0.7 for 41 SNPs within the Choquet summary statistics, totaling 564 SNPs [37]. In total, including the 47 lead SNPs from the Choquet meta-GWAS, we obtained 611 literature-derived variants containing summary statistic information [27]. Next, when a single study reported multiple significant dependent variants within a locus, we chose the most significantly reported variant. When multiple articles reported different and dependent variants in the same locus (LD threshold r^2^ ≥ 0.1), the variant from the study with the largest sample size was selected (while preserving the lead SNPs), resulting in 268 independent SNPs used for the glaucoma GRS [35, 37].

To obtain the glaucoma GRS, the individual SNP effect sizes were multiplied by the allelic dosages and summed across all 268 SNPs (Eq. 1) for all participants. Individuals at the highest and lowest 20% of the genetic risk distribution are invited to participate in EyeLife.

### Data gathering, interpretation, and phenotyping

**Fig. 2:**
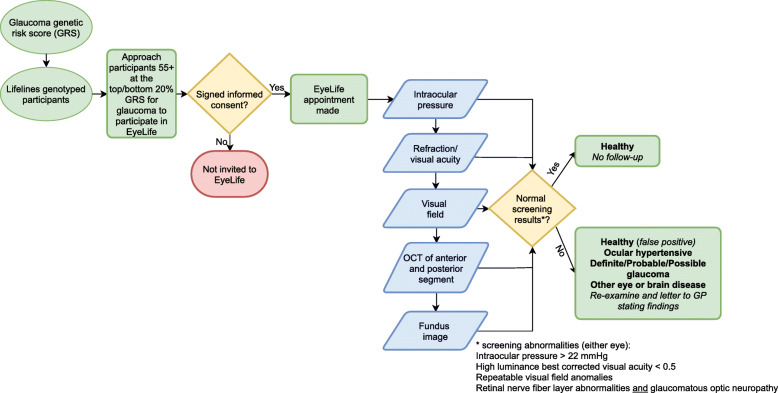
Flow diagram of EyeLife. The selection process, ocular examinations, and decision tree for EyeLife findings is described

EyeLife participants undergo a series of ocular examinations. Figure 2 presents a flow diagram of participant selection, the EyeLife visit, and visit outcomes. Examinations were chosen for being non-contact, non-mydriatic, rapid, robust, easy-to-operate, and of proven value in glaucoma screening/research in population-based settings.

First, IOP is measured with the non-contact Ocular Response Analyzer (ORA; Reichert Ophthalmic Instruments, Inc., Buffalo, NY, USA) [38]. IOP is measured in both eyes; the measurement is repeated until the quality metric, wavefront score (WS), is above 6, with a maximum of two measurements per eye. We record the corneal-compensated IOP (IOPcc), corneal hysteresis (CH), and the Goldmann-correlated IOP (IOPg) of the measurement with the higher WS value.

Next, participants’ visual acuity and refraction are obtained via the Nidek ARK-1S (NIDEK CO., LTD., Gamagori, Japan) [39]. Autorefraction is an average of three measurements, and is followed by visual acuity measured in three luminance conditions: high luminance (200 cd/m^2^; best corrected visual acuity [BCVA]), low luminance acuity (10 cd/m^2^), and acuity with a built-in glare source. The device also obtains pupil diameter and keratometry.

The visual field is tested using the Zeiss Humphrey frequency doubling technique (FDT) with the C-20-1 screening mode (Carl Zeiss Meditec, Jena, Germany) [40–44]. The test is repeated with any abnormal test location *P* < 0.01. Visual field loss (VFL) is defined as the presence of one or more reproducibly abnormal test locations. Then GVFL is defined as the presence of VFL that is not explained by abnormalities in the fundus images, and excluding homonymous or bitemporal defects.

Following, the structural layers of the eye are taken via the Optopol OCT, NX700 (OPTOPOL technology, Zawiercie, Poland). We image the optic disc region, the macular area, and the anterior chamber including the cornea. For the optic disc region, we focus on the peripapillary RNFL (pRNFL); aberrations in the pRNFL are defined as clock-hour defects at 1% of age-, gender-, and ethnicity-based normative data in the inferior, superior, or temporal clock-hours [45]. Central corneal thickness (CCT) is measured via an anterior segment OCT scan and an averaged value of both eyes is obtained.

Finally, fundus photographs are obtained via the Nidek AFC-330 (NIDEK CO., LTD., Gamagori, Japan) where two 45-degree angle-of-view images are taken of both eyes, one centered on the optic disc and one centered on the macula. The optic disc is assessed from fundus images by a glaucoma specialist (NJ). Glaucomatous optic neuropathy (GON) is defined as either a vertical cup-disc ratio (VCDR) of at least 0.7 (the 97.5th percentile in the general population), [46] or notching (focal absence of the neural rim) inferiorly or superiorly, or the presence of a peripapillary splinter haemorrhage [46–48].

We invite participants for re-examination by an ophthalmologist with at least one of the following findings: (1) IOPcc > 22 mmHg in either eye; (2) high luminance BCVA < 0.5 in either eye, unless there is a clear history of amblyopia or the participant is being treated for age-related macular degeneration; (3) VFL in either eye; and (4) RNFL abnormalities together with GON.

### Glaucoma classification

The International society of Geographical and Epidemiological Ophthalmology (ISGEO) classification is used as an initial reference point in defining glaucoma within EyeLife [48]. We classify EyeLife participants as definite, probable, or possible glaucoma, or healthy, similar to the Rotterdam Study [48]. We made two modifications to the ISGEO classification system. First, we removed the second GON cut-off point, the 99.5th percentile, for statistical reasons [49]. Second, currently RNFL assessment by OCT or other techniques is not within the ISGEO classification system, thus we modified classification according to the Northern Finland birth cohort eye study. They employed a ‘two-of-three’ (VF, RNFL, optic disc) method to classify glaucoma [50]. The resultant glaucoma categories are:

*Definite glaucoma* (combined functional and structural loss; the equivalent of ISGEO Category 1 diagnosis): GVFL with either RNFL abnormalities or GON;

*Probable glaucoma* (functional or confirmed structural loss): either GVFL (called ‘field suspect’ by the ISGEO) or RNFL abnormalities together with GON;

*Possible glaucoma* (structural loss): either RNFL abnormalities or GON (called ‘disc suspect’ by the ISGEO).

A glaucoma “case” is then defined as either *definite* or *probable* glaucoma. Missing data due to, for example, small pupils or media opacities, are interpreted as “normal findings” with other ocular tests examined in this context. For example, given the classifications above, a participant with GVFL, a normal fundus image, and missing OCT data is classified as probable glaucoma, based on the GVFL. Glaucoma is considered *open-angle glaucoma* (OAG) if an open-angle was found during gonioscopy at the re-invite (trabecular meshwork visible in at least 3 of 4 quadrants without compression); OAG was considered *primary OAG* (POAG) if there were no signs of pigment dispersion or pseudoexfoliation, or (a history of) other eye diseases that may have caused glaucoma. Intraocular pressure is not taken into account in the glaucoma diagnosis, however an IOPcc > 22 mmHg during the initial screening alongside an applanation tonometry (AT) reading above 20 mmHg during the re-invite is defined as *ocular hypertension* (OHT), if no GVFL, RNFL abnormalities, or GON is present. Although CCT is not taken into account in the glaucoma or OHT diagnosis, it is considered in clinical follow-up. A clinical follow-up (via GP referral) is offered to those with definite glaucoma, or in cases of reproducibly elevated IOP (IOPcc > 22 mmHg and AT > 20 mmHg) with or without probable or possible glaucoma. We only refer OHT cases with CCT ≥ 600 μm in combination with AT > 25 mmHg.

### Sample size

Assuming an odds ratio of 3.5, based on a published odds ratio for family history, [51] and a total population prevalence of glaucoma of 3.2% [46] for our target group of participants aged 55 plus (the prevalence in the top 20% genetic risk distribution of 5.0% and a prevalence of 1.5% in the bottom 20% genetic risk distribution), a power of 0.80, and an alpha of 0.008 we will be able to detect differences as small as 0.15 standard deviations for continuous traits with 660 subjects per risk group, which are clinically relevant effect sizes for glaucoma endophenotypes of interest; IOP, CCT, RNFL, and optic disc parameters (power calculator PASS11; Hintze, J. (2011). PASS 11. NCSS, LLC. Kaysville, Utah, USA. www.ncss.com.) [25]. With an assumed drop-out rate of 20%, we will invite 800 participants per risk group. Given the above assumptions, we estimate approximately 33 glaucoma cases (where a case is either *definite* or *probable* glaucoma) will be found in the participants constituting the top 20% of the genetic risk distribution for glaucoma, and nine cases in those at the bottom 20% of the genetic risk distribution.

### Selection, blinding, and minimization of bias

Using the glaucoma GRS, we identified 8279 Lifelines participants at the top, and 8199 participants at the bottom 20% of the genetic risk distribution for glaucoma. Subsequently, Lifelines refines this list based on age (55+), proximity to the research centre (postcodes 9300–9999), and participation status (if the participant is alive, still actively participating in Lifelines research, and amenable to additional studies). Lifelines sends this refined list of selected eligible participants to a third party to draw 800 participants at random from the top 20% of the genetic risk distribution and 800 from the bottom 20%. Participants are anonymized with a unique EyeLife ID. During the visit only the EyeLife ID, age, gender, and ethnicity of the participant is recorded.

In order to minimize bias, researchers, data collectors and participants were blinded to the genetic profile of participants throughout data collection. Any associations between genetic profile and outcome of the glaucoma assessment will be analysed after data collection is completed.

### Data analysis

The primary outcome is the relative risk of glaucoma (definite or probable) given a high genetic risk, see Table 1.

**Table 1:** Data outcome table of genetic risk and glaucoma of EyeLife

	High genetic risk	Low genetic risk	Total
Glaucoma	a	c	a + c
No glaucoma	b	d	b + d
Total	a + b	c + d	a + b + c + d

The glaucoma relative risk (RR) is then:

2 $$ RR=\frac{a/\left(a+b\right)}{c/\left(c+d\right)}. $$

Following, the standard error (SE) of the log to the base *e* of the relative risk is then:

3 $$ \mathrm{SE}\left\{\ln \left(\mathrm{RR}\right)\right\}=\sqrt{\frac{1}{a}+\frac{1}{c}-\frac{1}{a+b}-\frac{1}{c+d},} $$

and the associated 95% confidence interval (CI):

4 $$ 95\% CI=\mathit{\exp}\left(\mathit{\ln}(RR)-1.96\ast SE\left\{\mathit{\ln}(RR)\right\}\right)\kern0.5em to\kern0.50em \mathit{\exp}\left(\mathit{\ln}(RR)+1.96\ast SE\left\{\mathit{\ln}(RR)\right\}\right) $$

The secondary outcomes are to assess if glaucoma endophenotypes (IOP, DA, CA, CDR, CCT, and RNFLT) are significantly different between high and low glaucoma genetic risk distribution.

## Discussion

As the awareness of glaucoma status hovers at approximately 50%, an effective population-based screening procedure for glaucoma will enable earlier diagnoses and treatment thus preserving ocular health. There is an increased interest in the explanatory power of genetic variants to disease risk, as those at the higher end of the genetic risk distribution could be more intensively monitored, i.e. personalized screening, affording early detection and prevention. A recent report investigated the power of glaucoma detection with GRS that include endophenotypes in a case-control cohort [26, 52]. Although the explanatory power of a glaucoma genetic risk score is promising, thus far it is applied retrospectively. This study employs a glaucoma GRS prospectively to investigate its promise and feasibility as a pre-screening tool.

If this method is found to be an effective pre-screening approach for glaucoma in a population-based cohort, the next steps include optimizing the GRS with up-to-date SNPs implicated with glaucoma risk, including relevant endophenotypes, confirming our findings in a second population-based cohort, and extending the approach to different ethnic groups. The eventual goal is to detect glaucoma cases as early as possible to maintain eyesight for as long as possible.

## Data Availability

The data that support the findings of this study are available from Lifelines but restrictions apply to the availability of these data, which were used under license for the current study, and so are not publicly available. Data are however available from the authors upon reasonable request and with permission of Lifelines.

## Abbreviations

AT: Applanation tonometry
BCVA: Best corrected visual acuity
CCT: Central corneal thickness
CI: Confidence interval
IOPcc: Corneal compensation IOP
Ch: Corneal hysteresis
CA: Cup area
CDR: Cup-to-disc ratio
DA: Disc area
FDT: Frequency doubling technique
GRS: Genetic risk score
GWAS: Genome-wide association study
GON: Glaucomatous optic neuropathy
GFVL: Glaucomatous visual field loss
GSA: Global Screening Array
IOPg: Goldmann-correlated IOP
ISGEO: International society of Geographical and Epidemiological Ophthalmology
IOP: Intraocular pressure
LD: Linkage disequilibrium
OHT: Ocular hypertension
ORA: Ocular Response Analyzer
OAG: Open angle glaucoma
ONH: Optic nerve head
OCT: Optical coherence tomography
PPA: Peripapillary atrophy
pRNFL: peripapillary RNFL
POAG: Primary open angle glaucoma
RR: Relative risk
RNFL: Retinal nerve fiber layer
SNP: Single nucleotide polymorphism
SE: Standard error
SOP: Standard operating procedure
UGLI: UMCG Genetics Lifelines Initiative
UMCG: University Medical center Groningen
VCDR: Vertical cup-disc ratio
VF: Visual field
VFL: Visual field loss
WS: Wavefront score
